# The FAST D protocol: a simple method to rule out traumatic vascular injuries of the lower extremities

**DOI:** 10.1186/s13089-017-0063-2

**Published:** 2017-03-21

**Authors:** Miguel Angel Montorfano, Lisandro Miguel Montorfano, Federico Perez Quirante, Federico Rodríguez, Leonardo Vera, Luca Neri

**Affiliations:** 1 Department of Ultrasound, Clemente Álvarez Emergency Hospital, Rosario, Argentina; 2 AREU EMS Public Regional Company, Niguarda Ca’ Granda Hospital, Milan, Italy; 3 Av. Pellegrini 3205, 2000 Rosario, Santa Fe Argentina

**Keywords:** Doppler sonography, Critical ultrasound, Point-of-care ultrasound, Vascular injury, Lower extremity, Gunshot wounds, Trauma, FAST

## Abstract

**Background:**

The aim of this study is to assess the accuracy of a Fast Doppler protocol for the examination of an injured lower limb, namely 2-Point Fast Doppler (2PFD), in order to rapidly triage arterial lesions after penetrating trauma.

**Methods:**

The presence of flow and the aspects of the Doppler waveform of the dorsalis pedis artery (DPA) and posterior tibial artery (PTA) of the injured lower limb (2PFD) were evaluated immediately before the execution of a standardized Color Duplex Doppler (SD) evaluation in 149 limbs of 140 patients with gunshot penetrating injuries. We considered 2PFD normal exams as the ones with triphasic patterns in both the DPA and PTA, and 2PFD pathologic exams as the ones with absent, biphasic, or monophasic flow patterns in the DPA and/or PTA. 2PFD data were then analyzed to assess accuracy variables, using SD results as matching test reference. According to the trauma center standard protocols, SD positive cases underwent also angiography and surgical exploration, whose findings were used to further match the 2PFD specificity.

**Results:**

The 2PFD protocol showed a sensitivity of 100%, and a specificity of 100% compared with the SD, in the diagnostic workup of arterial injuries of the lower limbs after penetrating trauma. Furthermore, all the pathologic cases that resulted in all true positives (TP), compared with SD, were confirmed as TP also when matched with the angiography evaluation results.

**Conclusions:**

The 2PFD protocol can rapidly identify arterial flow and differentiate between normal and pathologic spectral Doppler analyses in distal arteries. The presence of the normal triphasic flows in DPA and PTA is as sensitive as the standardized Color Doppler Duplex assessment of the entire limb in ruling out arterial lesions in lower-limb penetrating trauma. The absence of flow or the presence of a biphasic or monophasic pathologic flow in DPA and PTA is pathologic and should be always followed by further investigation. 2PFD is faster and easier to perform compared with the SD approach. It could become a new first-line screening technique, both in pre-hospital and hospital critical scenarios, particularly in contexts where advanced diagnostic performance is limited by time concerns or scarce resources.

## Background

**Fig. 1:**
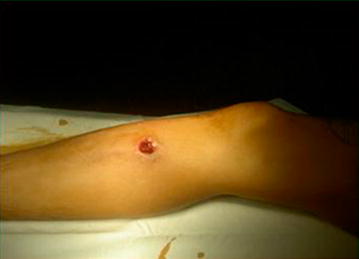
Gunshot injury

Gunshot wounds represent a major public health problem, with rising morbidity, mortality, and healthcare costs in many countries worldwide [1–7]. They are mainly caused by urban violence and account for the majority of penetrating vascular injuries in the civilian population. Many of these injuries are found in the lower limbs [8–10] (Fig. 1).

The primary evaluation and detection of significant vascular injury rests in the physical examination. The presence of the so-called “hard signs” (such as pulsatile external bleeding, expanding hematoma, absent distal pulses, cold/pale limb, palpable thrill, or audible bruit) mandates immediate intervention, particularly when the site of injury is obvious and the patient is hemodynamically unstable.

‘Soft signs’ of vascular injury (such as peripheral nerve deficit, history of moderate hemorrhage at scene, a reduced but palpable pulse, or an injury in proximity to a major artery) require instead further imaging investigation and observation, particularly when the site of vascular injury is less obvious (high-velocity weapons, multiple fragment injuries, and blunt trauma) and the patient is stable.

In stable patients without hard signs of vascular lesion, angiography of the lower extremities is considered the gold standard imaging method for the diagnosis of vascular injury after trauma. Yet, it is unavailable in certain scenarios, such as trauma scenes, mass casualty situations, and remote places.

Standardized Color Doppler Duplex ultrasound is a noninvasive examination combining B-mode and Doppler ultrasound widely available and can be used to obtain essential information for the diagnosis of these lesions [11–14]. Due to its highest sensitivity for limb artery injuries (Sn 95–97%), SD is presently considered a first-line examination for triaging such injuries, replacing conventional angiography in ruling out significant lesions. Although it may be less sensitive than angiography in the detection of clinically insignificant lesions such as small intimal defects or small vessel occlusions, it is as sensitive in detecting more significant lesions such as major intimal tears, pseudo-aneurysms, arteriovenous fistulas, thrombosis, and major vessel occlusions. SD is also considered highly specific (Sp 95–98%); however, when lesions are detected, angiography is also usually performed before any surgery, to better define the lesion itself and map the entire limb arterial and venous system. Nonetheless, the implementation of this technique on a large scale is limited by the fact that it is time consuming and quite operator-dependent.

Point-of-care ultrasound (POCUS) has been used to deliver fast answers to focused life-threatening and/or goal-directed clinical questions. Over the last decade, it has been increasingly used across a large number of specialties and scenarios [15–17], as it can be delivered in a timely and accurate manner, also by nonimaging specialists, with portable and/or more accessible equipment.

To our knowledge, there are no reports in the literature regarding the use of focused Doppler ultrasound to detect vascular injuries after penetrating trauma of the lower extremities. The aim of this study is to assess the accuracy of a rapid Doppler ultrasound evaluation of two distal arteries of the lower limbs, such as TPA and DPA, in order to rule out or rule in vascular injuries after penetrating trauma.

## Methods

From February 2011 to December 2015, a prospective diagnostic test accuracy study was conducted at a level 1 trauma center in Rosario, Santa Fe, Argentina. The ultrasound department has a 4-year ultrasound residency program and performs over twenty-five thousand ultrasounds per year. The internal review board of the hospital approved this study and waived the requirement for informed consent.

All patients who presented with gunshot injuries in the lower limbs were considered for this study, with the exception of all the patients who were hemodynamically unstable and were taken directly to the operating room without further imaging diagnostic workout and those without follow-up.

As per standard of care, in this trauma center, a standardized full Color Doppler Duplex ultrasound of both limbs (SD) is routinely performed to all patients admitted for gunshot wounds of the extremities, who are hemodynamically stable, when there is clinical suspicion of vascular injury. For the purpose of this study, a two-point Doppler assessment of the TPA and DPA (2PFD) was immediately done before performing the SD.

All the studies were performed by two Board-certified medical doctors, specialists in ultrasound and Doppler (MM, VL). The official diagnosis was established in the hospitalization report. Both procedures were never performed by the same attending physician, and they were blinded to the previous results.

A specific data collection form was used. The 2PFD results were compared with the SD outcome data. All pathologic cases (SD+) underwent confirmatory computed tomography angiography or arteriography and went on to further surgical treatment.

### Ultrasound approach

All Doppler ultrasound studies were performed with a broadband linear array transducer with a frequency range of 5–12 MHz (Toshiba Xario; Tokyo, Japan). Patients were scanned in a supine position. Transverse and longitudinal scans of the vessels were performed. The equipment was set with a depth of 2–3 cm to scan DPA and TPA, and with a depth of 5–6 cm to scan the proximal thigh (femoral artery, superficial femoral artery and deep femoral artery) depending on the body habitus of the patient. Two types of Doppler ultrasound modality were used. First, a color flow Doppler (CFD) imaging that showed the mean flow velocity distribution (displayed as a color-encoded map superimposed on the gray-scale B-mode tissue image), was used to recognize the arteries, both in transverse and longitudinal sections. Then, a spectral Doppler image showing the time-varying flow velocity distribution within a selected sample volume (PWD) was done only in longitudinal sections. To obtain reproducible information from PWD, the velocity scale was set between 10 and 60 cm per second, and a Doppler angle of insonation equal to or less than 60° was used.

**Fig. 2:**
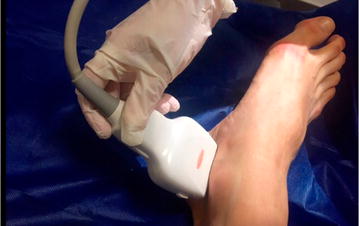
Posterior tibial artery scan

**Fig. 3:**
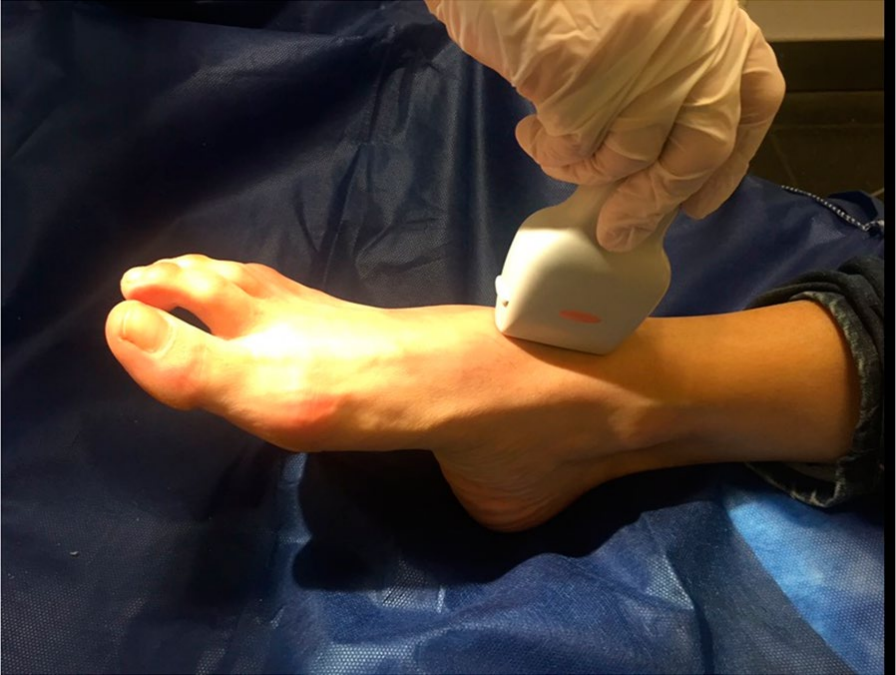
Dorsalis pedis artery scan

**Fig. 4:**
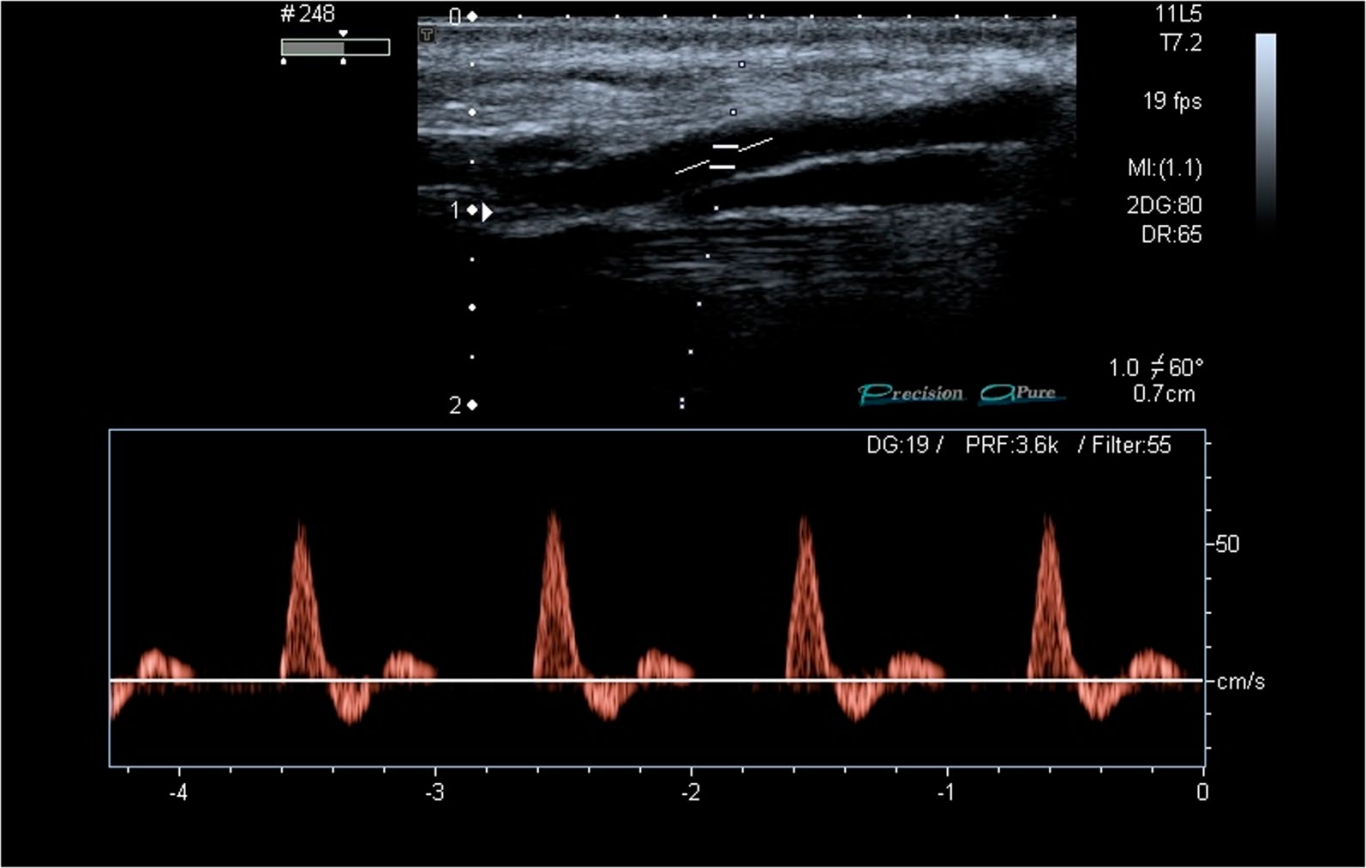
Triphasic Duplex waveform

**Fig. 5:**
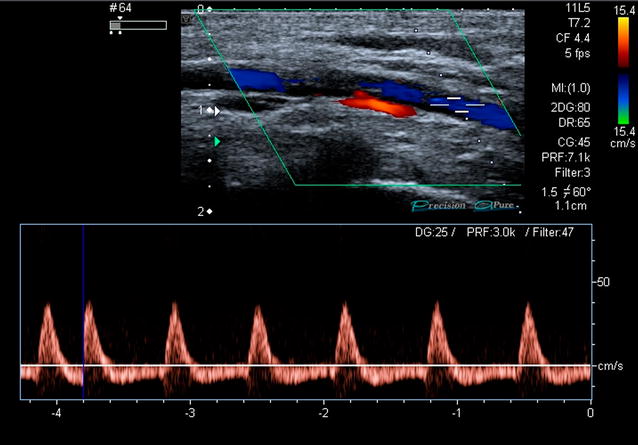
Biphasic Duplex waveform

**Fig. 6:**
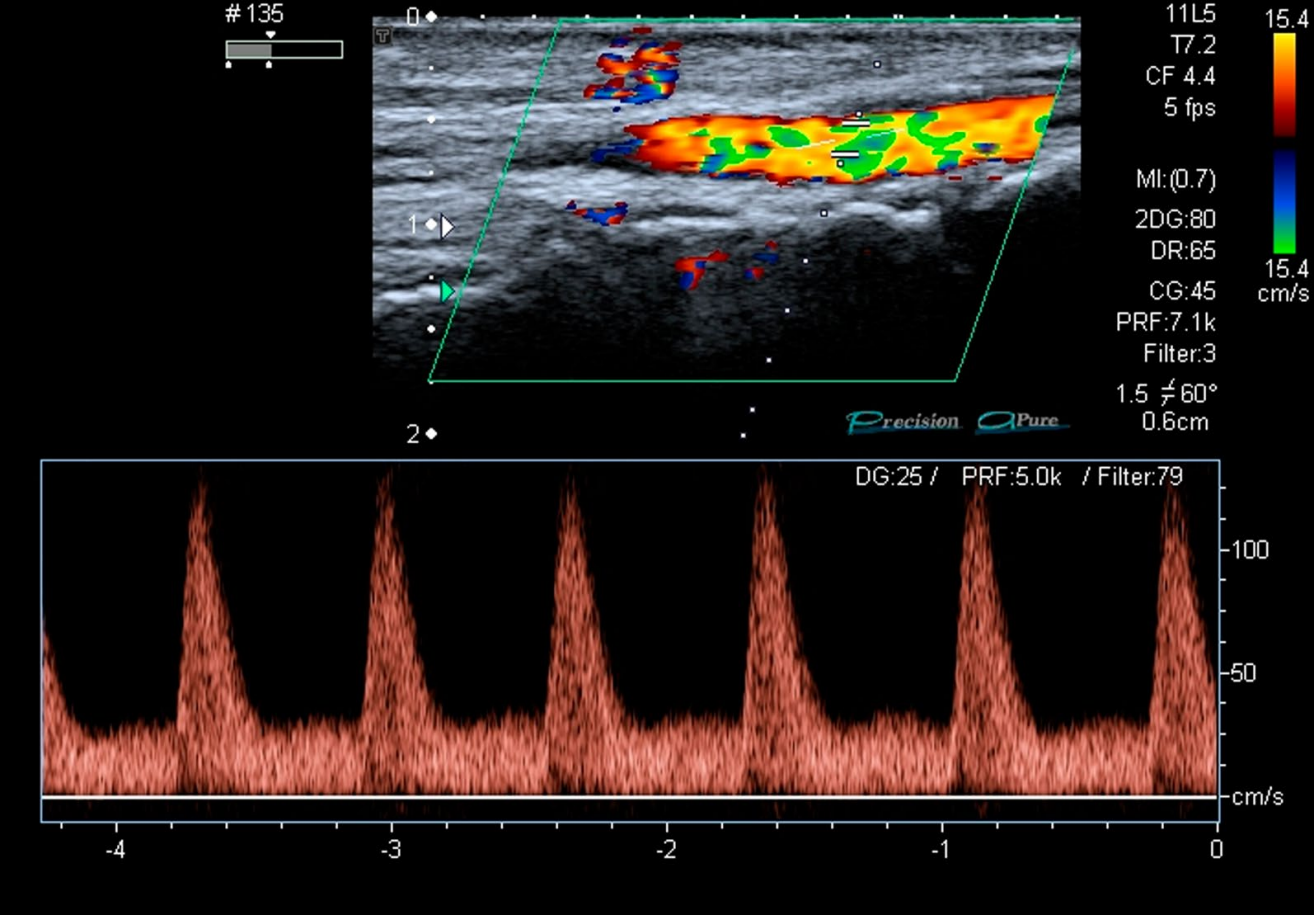
Monophasic Duplex waveform

According to the newly designed 2PFD protocol, we scanned two specific regions. First, the probe was placed posterior to medial malleolus of the tibia to evaluate the PTA flow (Fig. 2). Second, the probe was placed on the anterior part of the ankle to assess the flow of the DPA (Fig. 3). We considered normal (2PFD−) the presence of triphasic waveform with a narrow spectral width throughout the pulse cycle in both areas (Fig. 4). We defined as pathologic the absence of flow or the presence of biphasic or monophasic waveforms (2PFD+) in at least one of those arteries (Figs. 5, 6).

In the SD examination, we evaluated the entire arterial system of the injured lower limb, including the dorsalis pedis, posterior tibial, fibular, anterior tibial, popliteal, femoral, superficial femoral, deep femoral and iliac arteries.

## Results

Out of all the studied population assessed for gunshot injury at our institution from 2011 to 2015, 140 patients met the inclusion criteria. Out of these, 131 patients had an injury just in one limb and nine patients in both limbs, and thus, both 2PFD and SD examinations were performed over 149 injured lower limbs. Males represented the vast majority of our patient population (95%). Mean age was 27.15 years (STD = 10.64).

Injured lower limbs (149) were first screened with the 2PFD technique. We considered pathologic/positive exams (2PFD+), injured limbs with PDA and/or PTA presenting absent, biphasic or monophasic waveforms, and normal/negative exams (2PFD−) those ones with a triphasic flow pattern in both arteries. According to the results, the injured limbs were categorized into two groups: Group A (*n* = 134 limbs) with nonpathologic results (2PFD−), and group B (*n* = 15 limbs) with pathologic findings (2PFD+).

Distributions among groups are presented in Table 1.

**Table 1:** Population distribution

	Overall patients, *N* = 140	Normal, *N* = 125	Pathologic, *N* = 15
One-limb injuries	94% (131)	93% (116)	100% (15)
Two-limb injuries	6% (9)	7% (9)	0% (0)
Sex: male	95% (133)	94% (118)	100% (15)
Age (years)	27.15 (STD = 10.64)	27.41 (STD = 10.92)	24.87 (STD = 7.43)

Numbers after percents are frequencies

*STD* standard deviation

After the 2PFD, all patients underwent a SD exam of the entire injured limb/s as a matching reference test.

The limbs where 2PFD detected normal triphasic blood flow both in DPA and PTA (defined as P2FD− and categorized as group A), in all cases showed also triphasic nonpathologic patterns during the SD test. Thus, 100% of the 2PFD− results can be considered True Negatives (TN), matching perfectly with SD− reference results. Moreover, we found that the limbs where 2PFD detected the absence of flow or the presence of biphasic or monophasic flow in the DPA and/or PTA (defined as 2PFD+, and categorized as group B), showed pathologic findings in all cases during the SD exam (SD+). Then also 100% of the 2PFD+ results have to be considered True Positives (TP), compared with SD+.

Within this specific sample of patients, we thus found that the 2PFD was 100% sensitive and 100% specific, and has 100% positive predicted value, compared with the reference results of the SD technique, respectively, in ruling out and ruling in vascular injuries of the lower limbs after penetrating trauma.

According to the specific trauma center standard protocol, all the 15 SD+ patients/limbs (who were also 2PFD+), were further evaluated by angiographic techniques. Thirteen patients underwent computed tomography angiography and two underwent arteriography. In all cases, these studies confirmed the suspicion of arterial injury, as suggested both by the 2PFD and SD+, so that all the patients underwent surgical intervention. Comprehensive SD and surgical exploration finally reported, out of the 15 patients/limbs, a similar anatomic-pathologic type of diagnosis: 7 pseudo-aneurysms (47%), 5 direct injuries to the artery (33%), 2 arteriovenous fistulas (13%), and 1 artery compressing hematoma (7%). As collateral finding of the study, we can then highlight that using angiography and surgical exploration as reference exams, both 2PFD and SD proved to be 100% specific (i.e., 100% of TP) for vascular injuries detection; furthermore, SD proved to be 100% specific also for the evaluation of the type of injury.

With regard to the general distribution of entrance wounds along the lower limb, out of a total of 156 injuries throughout the 149 legs in 140 patients (seven patients had two gunshots in the same leg), we found 57% on the thigh (*n* 90), 26% on the leg (*n* 41), 7% on the knee (*n* 12), 4% on the inguinal region (*n* 6), 3% on the popliteal region (*n* 4), and 3% in the gluteal region (*n* 3). Further distribution is presented on Table 2. Regarding the specific distribution of entrance wounds among Doppler positive cases, 40% (6) were found on the thigh, 40% (6) on the leg, 13.3% (2) on the popliteal region, and 6.6% (1) in the gluteal region. All the pathologic cases had only one gunshot in the injured leg.

**Table 2:** Entrance wounds distribution

	Thigh	Lower leg	Knee	Inguinal region	Popliteal region	Gluteal region
Group A, *N* = 141	59% (84)	25% (35)	8% (12)	4% (6)	2% (2)	2% (2)
Group B, *N* = 15	40% (6)	40% (6)	0% (0)	0% (0)	13% (2)	7% (1)
Total, *N* = 156	57% (90)	26% (41)	7% (12)	4%(6)	3% (4)	2% (3)

Numbers after percents are frequencies

We only found exit wounds in 31% (*n* 49) of cases. Among the patients where we found an exit wound, 47% was through the thigh (*n* 23), 29% was through the lower leg (*n* 14), 8% was through the knee (*n* 4), 2% was through the inguinal region (*n* 1), 4% was through the popliteal region, and 10% were through the gluteal region (*n* 5). Regarding the specific distribution of exit wounds along the limb with positive Doppler exam, only 6.6% (*n* 1) were found in the thigh and 13.3% (*n* 2) in the leg. The rest of the pathologic cases did not have exit wounds.

## Discussion

Gunshot injuries are a substantial cause of vascular damage in the civilian population worldwide. Furthermore, they are associated with high morbidity and mortality [3–7]. Arterial limb injuries can result in extremity amputation or become a life-threatening scenario which demands immediate attention.

Angiography and surgical exploration have been defined in the literature as the gold standard approaches when the clinical suspicion for vascular injury is high [18]. Angiography is sometimes limited in critical scenarios or remote locations. Surgical exploration carries severe limitations as it is very resource dependent, and it is not always widely available for all patients. In catastrophe sites, for example, the physician has to carry out a triage assessment and decide who is in the most-urgent need of transportation to the operating room.

Less-invasive methods such as arterial pressure indexes and standardized Color Doppler Duplex ultrasound can be considered as valuable alternatives for the triage assessment. Burg et al. [19] reported an ankle-brachial index (ABI) of 0.9 or less as a sensitive test for the identification of vascular injury of the lower extremities. However, the palpation of the peripheral pulses can be difficult in some patients due to vasoconstriction, hypovolemia, and pain. SD exam of the extremities allows for diagnosis of vascular lesions with a high sensitivity. Knudson et al. [20] stated that SD exam was equal in sensitivity compared with angiography in detecting vascular injury with the advantage of being noninvasive. The pulsed Doppler spectral analysis recorded from a peripheral lower extremity artery has the feature of a triphasic waveform with a narrow spectral width throughout the pulse cycle, indicating red blood cells moving at a similar speed and direction in a nondisturbed or laminar flow pattern [21]. Any lesion, including arteriovenous fistulas, traumatic thrombotic arterial obstructions, pseudo-aneurisms, or external hematomas compressing the arteries, will impact distal flow, presenting abnormal patterns, such as monophasic, biphasic, or absent flow. Nevertheless, SD has also limitations, as it can be time consuming, and a properly trained radiologist is not always present in the emergency setting to perform the study (e.g.,, peripheral and/or resource-scarce hospitals, extra-hospital, or disaster scenarios).

Point-of-care ultrasound is a novel tool being increasingly used not only by the emergency physician but also by clinicians in several other specialties [15–17]. In the trauma setting, it allows physicians to make faster diagnostic and therapeutic decisions. A large volume of the literature describes how point-of-care ultrasound can be used to diagnose and manage a broad spectrum of conditions in critically ill patients [22–24]. In trauma patients, the evaluation of the extremities using focused ultrasound is limited nowadays to the diagnosis of fractures, soft tissues injuries, and the presence of foreign bodies [25, 26]. Yet, to our knowledge, this is the first report in the literature that describes how to rapidly rule out/in vascular injuries in patients who suffered from penetrating trauma of the lower extremities, by means of a 2-point Doppler technique.

The 2PFD protocol allows physicians to rapidly asses the presence or the absence of flow in two distal arteries of the lower limb, PDA, and PTA. Once this first assessment is done and flow is detected, the physician evaluates the waveform pattern (triphasic or biphasic/monophasic flow). In the absence of vascular injury, the spectral Doppler analysis normally shows a triphasic waveform over each cardiac cycle, representing the blood acceleration during systole: an early diastolic flow reversal caused by the closure of the aortic valve; and a late anterograde diastolic flow related with the elasticity of the arterial wall, peripheral resistance, and transmural gradient. In the presence of any kind of vascular damage or external compression (i.e., large hematoma), the physician could find no flow or the presence of either a monophasic or biphasic waveform pattern.

The 2PFD protocol has the advantage of being noninvasive and a rapid test, requiring no more than 2 min and probably easier to learn by nonimaging specialist, although learning curve analysis of this technique is a subject matter of further investigation.

The 2P Fast Doppler imaging technique can present yet some limitations. The absence of flow or a monophasic/biphasic Doppler Duplex pattern in distal arteries can be detected not only in acutely injured arteries, but also as result of chronic conditions, such as proximal diabetic angiopathy, severe atherosclerosis, or aging. This can cause misinterpretation of the results. As the 2PFD cannot differentiate if the pathologic flow is caused by an acute or a chronic lesion in positive cases, further investigation is always required. In our series, there were no diabetic patients, and the average age was 27 years (24.8 among the pathologic cases). Probably for this reason, there were no false positives related to these comorbidities in this study. As such comorbidities are generally related to older patients, the latter ones may represent a targeted population for more thorough examination.

In targeted patient populations, such as the “injured nondiabetic young patients” of our study, due to the low prevalence, or even the absence of concurrent chronic lesions (i.e., low or no false positives for vascular injuries), 2PFD could be used also as a triage tool to “rule in” acute cases with high specificity equal to SD (Sp 100%).

On another note, if the injured artery has no distal extension (i.e., profunda femoris artery = PFA), and there are no hematomas compressing other arteries, there will be no distal repercussion [27]. In this particular case, the FAST Doppler protocol would not show any abnormal distal pattern, leading to potentially false negative results. We did not have any PFA injuries in our study so this limitation did not affect the sensitivity of our findings. To overcome this limitation, when a penetrating injury of the supero-internal part of the thigh is present, further workup may still be warranted, and physicians would have to explore the thigh with point-of-care ultrasound and SD to identify if large hematomas, pseudo-aneurysms, or arteriovenous fistulas are present in each particular case [28]. In our hospital, we always explore the region injured by gunshot wounds in order to search not only for vascular lesions but also for bone fractures and soft tissue lesions.

In our study population, the 2PFD protocol has proven to be a triage technique as sensitive as SD (Sn 100%). In fact, our data show that the detection of triphasic waveforms can be a highly sensitive method in ruling out vascular injuries of the lower extremities after penetrating trauma.

**Fig. 7:**
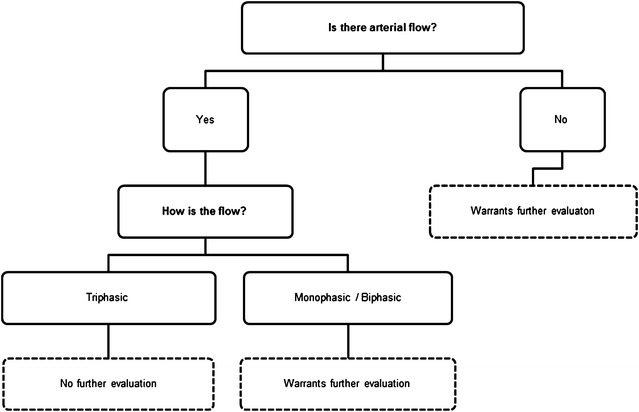
Fast D Protocol

The absence of flow or the presence of a biphasic or monophasic pathologic flows in PDA and/or PTA must be considered pathologic and should be always followed by further investigation, such as SD, angiography, or surgical exploration, to confirm the proximal lesion, and define its type, extension, and distribution (Fig. 7).

## Conclusions

The 2-PFD protocol is a focused goal-directed Doppler procedure that could be used both in pre-hospital and hospital critical settings, as a triage tool capable to “rule out” arterial injuries in lower-limb penetrating trauma, with the highest sensitivity comparable with standard Doppler examination and angiography.

In particular clusters of patients, where the prevalence of chronic vascular lesions is negligible (e.g., nondiabetic young subjects), this focused technique could be also considered a reliable tool to “rule in” acute lesions with high specificity.

However, as the 2PFD cannot differentiate if the pathologic flow is cause by an acute or a chronic lesion, in positive cases, further immediate investigation and observation is always required.

In the future, if such an accuracy will be confirmed, the 2PFD protocol could become part of a broader FAST Doppler integrated protocol for the primary assessment of the trauma patient, particularly in contexts where the diagnostic and management performance is limited by time concerns (unstable and/or multi-injured patients, mass casualties, combat, or other risky settings) or scarce resources (poor technology and/or competences, and remote/rural/austere settings), enhancing the decision-making process and allowing for better allocation of resources and improved patient care [29].
